# Gram-positive bacteria cell wall-derived lipoteichoic acid induces inflammatory alveolar bone loss through prostaglandin E production in osteoblasts

**DOI:** 10.1038/s41598-021-92744-5

**Published:** 2021-06-25

**Authors:** Tsukasa Tominari, Ayumi Sanada, Ryota Ichimaru, Chiho Matsumoto, Michiko Hirata, Yoshifumi Itoh, Yukihiro Numabe, Chisato Miyaura, Masaki Inada

**Affiliations:** 1 Department of Biotechnology and Life Science, Tokyo University of Agriculture and Technology, 2-24-16 Nakacho, Koganei, Tokyo 184-8588 Japan; 2 Cooperative Major of Advanced Health Science, Tokyo University of Agriculture and Technology, 2-24-16 Nakacho, Koganei, Tokyo 184-8588 Japan; 3 Institute of Global Innovation Research, Tokyo University of Agriculture and Technology, 2-24-16 Nakacho, Koganei, Tokyo 184-8588 Japan; 4 Nuffield Department of Orthopaedics, Rheumatology and Musculoskeletal Sciences, Kennedy Institute of Rheumatology, University of Oxford, Oxford, OX3 7FY UK; 5 Department of Periodontology, School of Dentistry, The Nippon Dental University, 1-9-20 Fujimi, Chiyoda-ku, Tokyo, 102-0071 Japan

**Keywords:** Molecular biology, Diseases

## Abstract

Periodontitis is an inflammatory disease associated with severe alveolar bone loss and is dominantly induced by lipopolysaccharide from Gram-negative bacteria; however, the role of Gram-positive bacteria in periodontal bone resorption remains unclear. In this study, we examined the effects of lipoteichoic acid (LTA), a major cell-wall factor of Gram-positive bacteria, on the progression of inflammatory alveolar bone loss in a model of periodontitis. In coculture of mouse primary osteoblasts and bone marrow cells, LTA induced osteoclast differentiation in a dose-dependent manner. LTA enhanced the production of PGE_2_ accompanying the upregulation of the mRNA expression of mPGES-1, COX-2 and RANKL in osteoblasts. The addition of indomethacin effectively blocked the LTA-induced osteoclast differentiation by suppressing the production of PGE_2_. Using ex vivo organ cultures of mouse alveolar bone, we found that LTA induced alveolar bone resorption and that this was suppressed by indomethacin. In an experimental model of periodontitis, LTA was locally injected into the mouse lower gingiva, and we clearly detected alveolar bone destruction using 3D-μCT. We herein demonstrate a new concept indicating that Gram-positive bacteria in addition to Gram-negative bacteria are associated with the progression of periodontal bone loss.

## Introduction

Periodontitis is a local infectious disease associated with inflammatory alveolar bone loss. Bacterial plaque in the periodontal pocket expands the growth and accumulation of Gram-negative bacteria, which induces inflammation in periodontal tissues including alveolar bone. Lipopolysaccharide (LPS) from Gram-negative bacteria is considered to be a dominant pathogen causing inflammatory bone resorption in periodontitis. We have established a mouse in vivo model of periodontitis caused by the local injection of LPS into the lower gingiva; the model shows prostaglandin (PG) E_2_ production and severe alveolar bone loss^1^. We have shown that alveolar bone loss could not be induced by LPS injection in membrane-bound PGE synthase (mPGES)-1 knockout mice, which suggests that the synthesis of mPGES-1-induced PGE_2_ is essential for the progression of periodontitis^1^.

Bone remodeling is precisely controlled by the balance between osteoclastic bone resorption and osteoblastic bone formation. Osteoclasts are differentiated from macrophage lineage cells and exhibit bone resorbing activity. The expression of receptor activator of NF-κB ligand (RANKL) on the cell surface of osteoblasts is essential for osteoclast differentiation^2–4^. Bone resorbing factors, such as pro-inflammatory cytokines enhance the RANKL expression in osteoblasts, and RANKL interacts with the RANK expressed on osteoclast precursor cells to lead the differentiation into mature osteoclasts^5,6^. PGE_2_ produced by osteoblasts is a well-known mediator in inflammatory bone resorption^7,8^. Inflammatory stimuli promote the activation and expression of key enzymes for PGE_2_ synthesis (cytosolic phospholipase A_2_ [cPLA_2_], cyclooxygenase [COX]-2 and mPGES-1). The release of arachidonic acid from membrane phospholipid is mediated by cPLA_2_, and COX-2 in turn converts arachidonic acid into PGH_2_, while mPGES-1 mediates the synthesis of PGE_2_ from PGH_2_. We have reported that LPS and pro-inflammatory factors, including interleukin-1α enhance the mPGES-1-mediated production of PGE_2_ and the RANKL expression in osteoblasts, leading to osteoclastogenesis and inflammatory bone resorption^1,8–10^.

Toll-like receptors (TLRs) play critical roles in the innate immune response, and various microbial components have been identified as ligands for respective TLRs. LPS, a well-known microbe-associated molecular pattern (MAMP) derived from Gram-negative bacteria in periodontitis, is a major cell-wall component of Gram-negative bacteria and identified as a ligand for TLR4. LPS-TLR4 signaling stimulates the PGE_2_-mediated RANKL expression, leading to subsequent inflammatory alveolar bone resorption^1^. TLR2 is known to recognize bacterial lipopeptide and form heterodimers with TLR1 or TLR6^11,12^. Lipoteichoic acid (LTA) is a major cell-wall component of Gram-positive bacteria associated with tooth decay and works as a ligand of TLR2/6 heterodimer^13–15^. It is well known that Gram-negative bacteria derived LPS dominantly induces inflammatory alveolar bone destruction; however, the roles of Gram-positive bacteria cell wall-derived LTA in periodontal bone resorption are remains unclear.

In this study, we examined the influence of LTA on osteoclast differentiation and bone resorbing activity. LTA promoted COX-2- and mPGES-1-induced PGE_2_ production in osteoblasts and acted on osteoclasts to prolong cell survival. LTA clearly induced inflammatory alveolar bone loss in a mouse model of periodontitis. Gram-positive bacteria may enhance inflammation in periodontitis with alveolar bone loss through the action of LTA.

## Results

### LTA induced osteoclast differentiation and bone resorption

**Figure 1:**
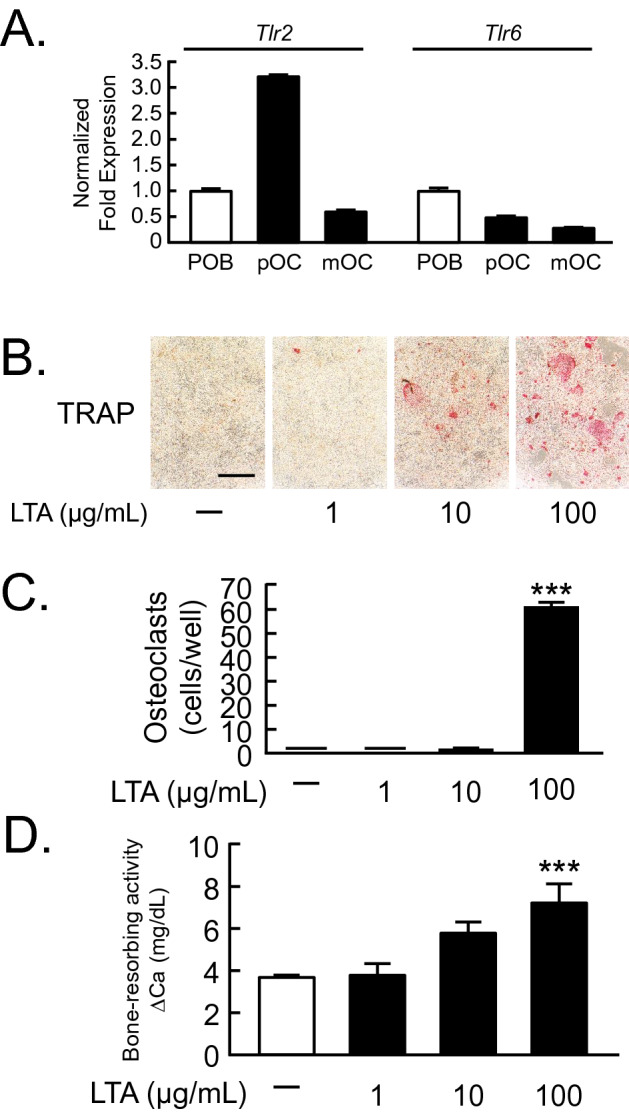
LTA induced osteoclast differentiation and bone resorption. (**A**) The mRNA expression of TLR2 and TLR6 in mouse primary osteoblasts (POBs), Raw264.7 cells (osteoclast precursor cells; pOC) and sRANKL-treated Raw264.7 cells (mature osteoclasts; mOC). The data are expressed as the means ± SEM of triplicate from a representative experiment. β-actin (*Actb*) was used as a normalized gene. (**B**) Mouse POB and BMCs were co-cultured with LTA (1, 10, 100 μg/mL) for 6 days. The images showed TRAP-positive multinuclear osteoclasts. Scale bar: 500 μm. (**C**) TRAP-positive multinuclear osteoclasts were counted. The data are expressed as the mean ± SEM of 3 wells. (**D**) Mouse calvariae from newborn mice were cultured with LTA (1, 10, 100 μg/mL) for 5 days. The calcium concentration was measured to elucidate bone-resorbing activity. The data are expressed as the mean ± SEM of 5 wells. A significant difference between the two groups was indicated; ***P < 0.001 versus control; by one-way ANOVA and Tukey’s post hoc test.

LTA is a ligand of TLR2/6 heterodimer, we first examined the mRNA expression of TLR2 and TLR6 in primary osteoblasts (POBs) and osteoclasts by RT-qPCR. Both TLR2 and TLR6 were expressed in POBs, Raw264.7 cells (osteoclast precursor cells [pOCs]) and sRANKL-treated Raw264.7 cells (mature osteoclasts [mOCs]) (Fig. 1A). In the coculture of bone-marrow cells (BMCs) and POB, LTA significantly induced osteoclast differentiation (Fig. 1B, C). To test the effect of LTA on bone resorption, mouse calvariae were cultured with LTA. The data indicated that LTA promoted bone-resorbing activity in calvarial organ cultures in a dose-dependent manner (Fig. 1D). Thus, LTA is a potent inducer of osteoclast differentiation and bone resorption.

### LTA stimulated the production of PGE_2_ via the upregulation of PGE_2_ synthesis-related genes in osteoblasts

**Figure 2:**
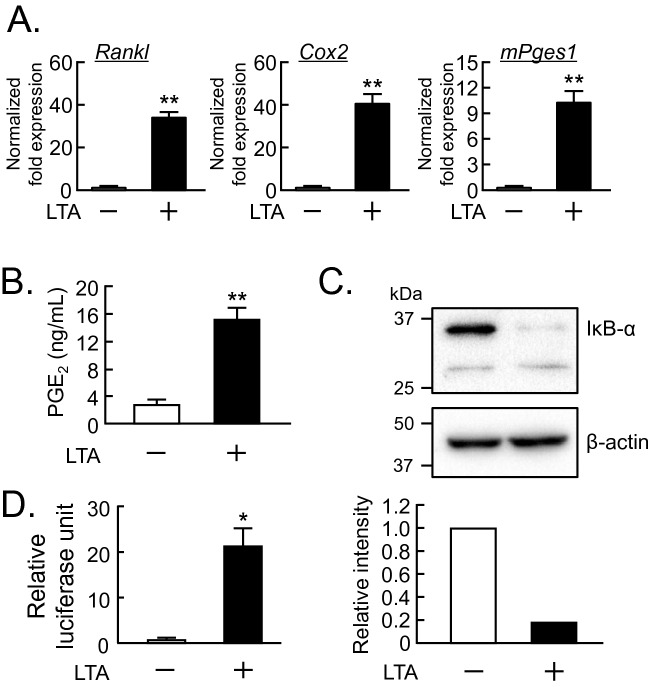
LTA promoted the production of PGE_2_ via the regulation of PGE_2_-related genes in osteoblasts. (**A**) Mouse POBs were cultured with LTA (100 μg/mL) for 24 h and total RNA was extracted, and the mRNA expression of RANKL, COX-2 and mPGES-1 was analyzed by qPCR. The data are expressed as the means ± SEM of triplicate from a representative experiment. β-actin was used as a normalized gene. (**B**) Mouse POBs were cultured with LTA (100 μg/mL) for 24 h, and the level of PGE_2_ in the conditioned medium was measured. The data are expressed as the mean ± SEM of 3 wells. (**C**) Mouse POBs were treated with LTA (100 μg/mL) for 15 min. Whole lysates were collected, and the protein level of IκBα was detected by Western blotting. Full-length blot images and blot images in the replicated experiment were shown in supplemental figures 1 and 2. (**D**) The transcription activity of NF-κB was measured with or without LTA (100 μg/mL). Plasmid pNFκB-TA-Luc (0.4 μg) and the pGL4.74[hLuc/TK] plasmid (40 ng) were transfected into POBs, and the luciferase activity was measured with the Dual-luciferase Reporter Assay system. The data are expressed as the mean ± SEM of 4 wells. A significant difference between the two groups was indicated; *P < 0.05 and **P < 0.01 versus control; by Welch’s *t-*test.

To clarify the mechanisms of osteoclast differentiation induced by LTA, we examined the effect of LTA on the mRNA expression of *Rankl*, *Cox2* and *mPges1* in POBs by RT-qPCR. LTA markedly upregulated the mRNA level of these genes in POBs (Fig. 2A). We further found that LTA induced the production of PGE_2_ by POBs (Fig. 2B).

We previously reported that LPS stimulated the transcriptional activity of NF-κB, leading to the increased mRNA expression of *Cox2* and *mPges1*^1^. To test whether LTA activates the NF-κB pathway, the protein level of IκBα, an endogenous inhibitor of NF-κB, was analyzed by Western blotting, since the activation of the NF-κB pathway by inflammatory molecules rapidly degrades IκBα via the ubiquitin–proteasome system. Adding LTA induced a notable reduction of IκBα (Fig. 2C) and promoted the transcriptional activity of NF-κB in a dual luciferase reporter gene assay (Fig. 2D).

### Indomethacin blocked LTA-induced bone resorption

**Figure 3:**
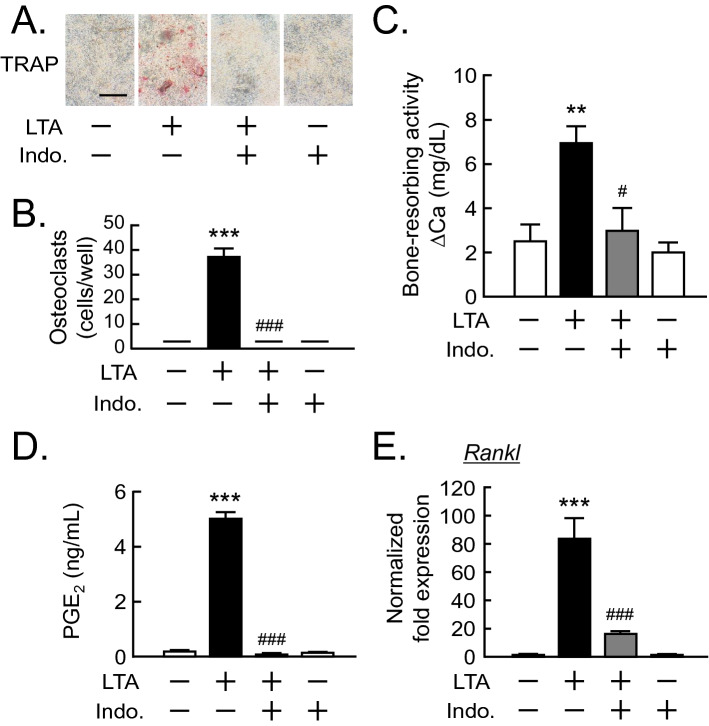
Effects of indomethacin on LTA-induced bone resorption. (**A**) Mouse POB and BMCs were co-cultured with or without LTA (100 μg/mL) and indomethacin (10 μM) for 6 days. The images showed TRAP-positive multinuclear osteoclasts. Scale bar: 500 μm. (**B**) TRAP-positive multinuclear osteoclasts were counted. The data are expressed as the mean ± SEM of 3 wells. (**C**) Mouse calvariae from newborn mice were cultured with LTA (100 μg/mL) and indomethacin (10 μM) for 5 days. The calcium concentration was measured to elucidate bone-resorbing activity. The data are expressed as the mean ± SEM of 5 wells. (**D**) Mouse POBs were cultured with LTA (100 μg/mL) and indomethacin (10 μM) for 24 h, and the level of PGE_2_ in the conditioned medium was measured. The data are expressed as the mean ± SEM of 3 wells. (**E**) Mouse POBs were cultured with LTA (100 μg/mL) for 24 h and total RNA was extracted and the mRNA expression of RANKL was analyzed by qPCR. The data are expressed as the means ± SEM of triplicate from a representative experiment. β-actin was used as a normalized gene. A significant difference between the two groups was indicated; **P < 0.01 and ***P < 0.001 versus control, ^#^*P* < 0.05 and ^###^*P* < 0.001 versus LTA; by one-way ANOVA and Tukey’s post hoc test.

To determine the roles of PGE_2_ in osteoclast differentiation, BMC and POB were cocultured with or without LTA and indomethacin, a typical nonsteroidal anti-inflammatory drug. Indomethacin markedly suppressed the LTA-induced osteoclast differentiation in the cocultures. (Fig. 3A, B). In calvarial organ cultures, indomethacin completely blocked the bone resorbing activity induced by LTA (Fig. 3C). In POBs, LTA-induced PGE_2_ production and the mRNA expression of RANKL were significantly blocked by the addition of indomethacin (Fig. 3D, E).

### LTA prolonged the life span of mature osteoclasts

**Figure 4:**
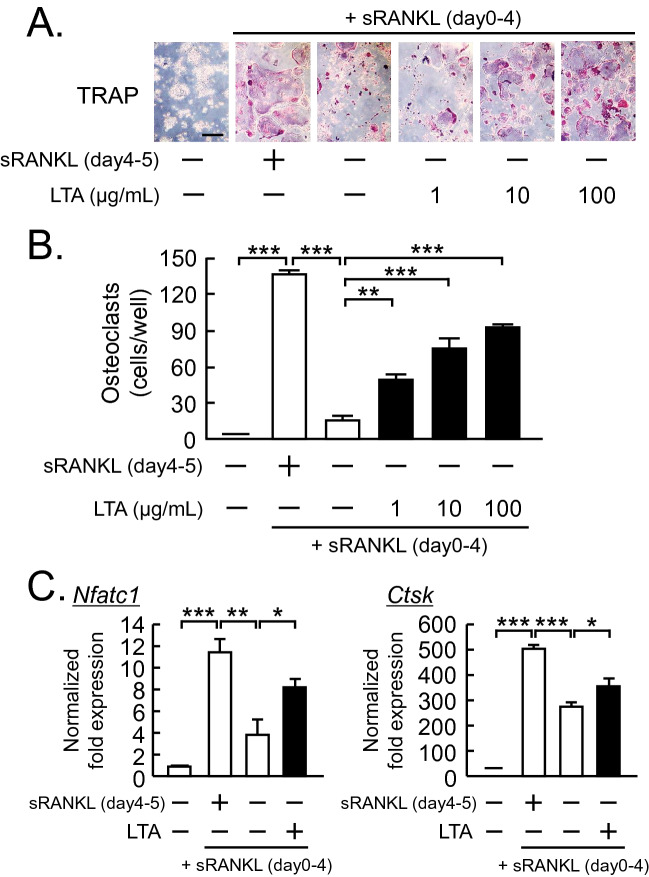
LTA enhanced the lifespan of mature osteoclasts. (**A**) Raw264.7 cells were cultured with sRANKL (100 ng/mL) for 4 days. After the formation of mature osteoclasts, osteoclasts were treated with LTA (1, 10, 100 μg/mL) in the absence of sRANKL for 1 day. The images showed TRAP-positive multinuclear osteoclasts. Scale bar: 200 μm. (**B**) TRAP-positive multinuclear osteoclasts were counted. The data are expressed as the mean ± SEM of 4 wells. (**C**) Osteoclasts were treated with LTA (100 μg/mL) or sRANKL (100 ng/mL). The mRNA expression of *Nfatc1* and *Ctsk* was analyzed by qPCR. The data are expressed as the means ± SEM of triplicate from a representative experiment. β-actin (*Actb*) was used as a normalized gene. A significant difference between the two groups was indicated; *P < 0.05, **P < 0.01 and ***P < 0.001; by one-way ANOVA and Tukey’s post hoc test.

We examined the effect of LTA on the lifespan of mOCs. Raw264.7 cells, a mouse macrophage cell line, were differentiated into mOCs by sRANKL, and the mOCs were cultured with sRANKL or LTA. The removal of sRANKL on day 4 induced the cell death of mOCs, while in the absence of sRANKL, LTA prolonged the lifespan of mOCs in a dose-dependent manner (Fig. 4A, B). It is well-known that NFATc1 (Nuclear factor of activated T-cells, cytoplasmic 1) encoded by the *Nfatc1* gene is the master transcription factor to osteoclast differentiation and survival, and cathepsin K encoded by the *Ctsk* gene is a major target gene of NFATc1 and relates to osteoclast activity^16^. To reveal the mechanism of LTA-induced osteoclast survival, we analyzed osteoclast marker genes, *Nfatc1* and *Ctsk*, by RT-qPCR. In the absence of sRANKL, the expression of *Nfatc1* and *Ctsk* in mOCs was increased by the addition of LTA (Fig. 4C).

### LTA induced alveolar bone loss in a mouse ex vivo model of periodontal bone resorption and in vivo model of periodontitis

**Figure 5:**
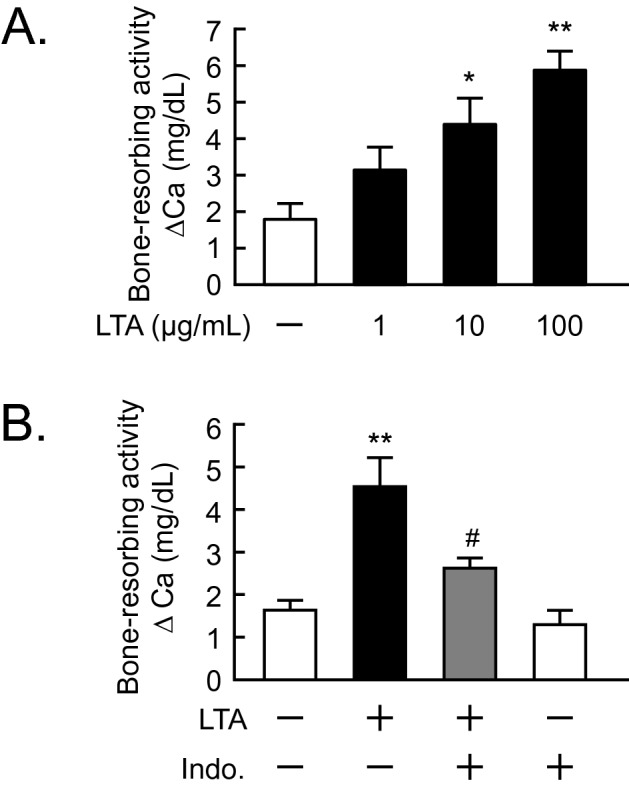
LTA induced bone resorption in an ex vivo mouse model of periodontal bone resorption for periodontitis. (**A**) Mouse alveolar bones were cultured with LTA (1, 10, 100 μg/mL) for 5 days. (**B**) Mandibular alveolar bones were cultured with LTA (100 μg/mL) in the presence or absence of indomethacin (10 μM) for 5 days. The calcium concentration was measured to elucidate bone-resorbing activity. The data are expressed as the mean ± SEM of 4 wells. A significant difference between the two groups was indicated; *P < 0.05 and **P < 0.01 versus control, ^#^*P* < 0.05 versus LTA; by one-way ANOVA and Tukey’s post hoc test.

We first examined an ex vivo model of periodontal bone resorption^1^. Mouse mandibular alveolar bone was collected and cultured with LTA for 5 days. LTA induced alveolar bone resorption was shown in a dose-dependent manner (Fig. 5A), while indomethacin significantly inhibited LTA-induced bone-resorbing activity (Fig. 5B).

**Figure 6:**
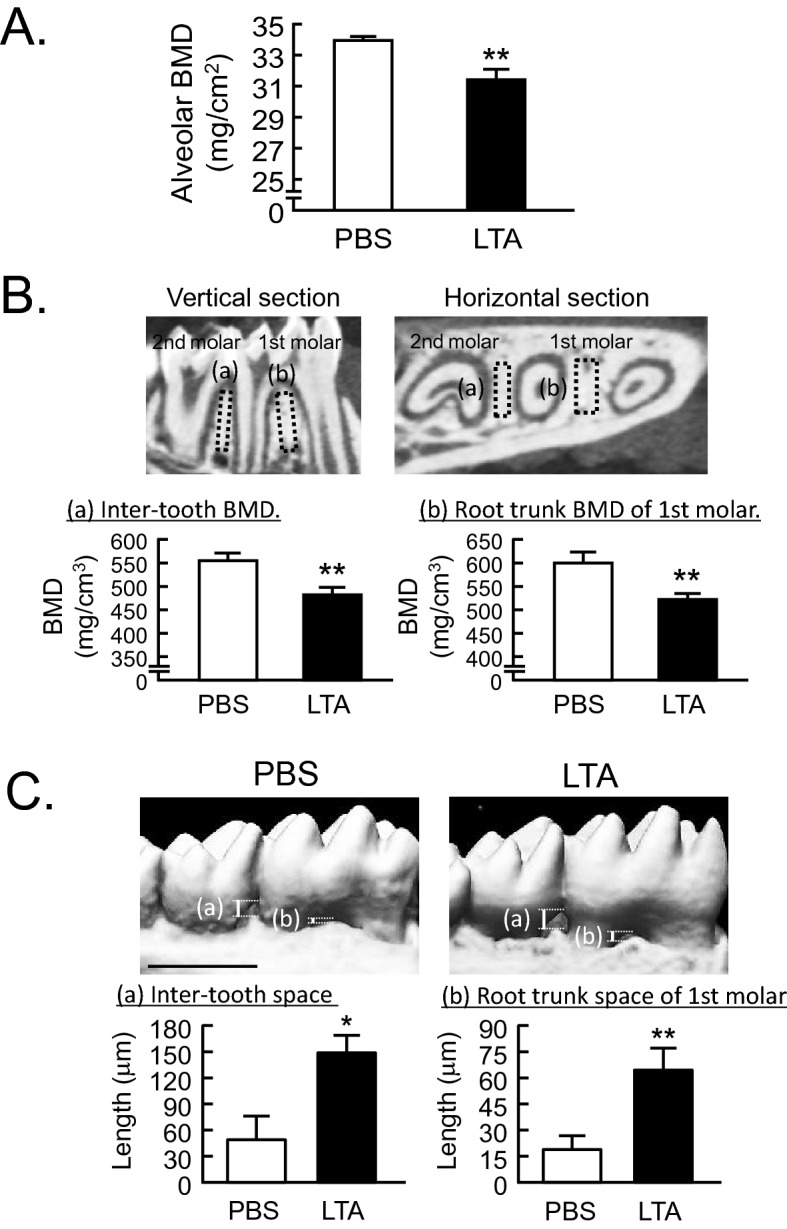
LTA induced bone resorption in an in vivo mouse experimental model of periodontitis. (**A**) The BMD of alveolar bone was measured using DXA. (**B**) The BMD of alveolar bone was calculated by an analysis of 3D-μCT imaging. The black dotted square indicates the analyzed area of the vertical and horizontal sections shown in the upper images. The BMD of the alveolar bone was measured in the inter-tooth areas of the 1st and 2nd molars (a), and at the root trunk of the 1st molar (b). Both areas showed a decreased bone mass, which reflected increased bone resorption by LTA. (**C**) The loss of vertical alveolar bone was analyzed by 3D-μCT imaging. The length of the vertical white line in the upper μCT images (a) indicates the length of alveolar bone loss from the gingival sulcus to the alveolar bone (inter-tooth space) and (b) indicates the bottom of the molar root trunk to the alveolar bone (root trunk space) of the 1st molar. The length reflected alveolar bone loss. Scale bar: 1 mm. The data are expressed as the mean ± SEM of 6 mice. A significant difference between the two groups was indicated; *P < 0.05 and **P < 0.01 versus control; by Student’s t-test.

We next examined the in vivo model of periodontitis with alveolar bone resorption^1^. In the mouse model of periodontitis, we injected LTA or PBS into the left outer gingiva of the first molar in the lower jaw at a depth of 1–2 mm on days 0, 2 and 4, and the bone mass of the alveolar bone was analyzed by dual-energy X-ray absorptiometry (DXA) and μCT on day 7. In the DXA analysis, alveolar BMD was significantly reduced by the injection of LTA (Fig. 6A). The alveolar BMD decreased by approximately 10%, PBS 34.0 mg/cm^2^ versus LTA 31.7 mg/cm^2^, a reduction that was similar to that noted following LPS injection in a mouse model of periodontal bone resorption^1^. Since the tooth root is exposed from the alveolar bone due to increased bone resorption, a 3D-μCT analysis is a definitive method for analyzing alveolar bone resorption in periodontitis^17^. We first examined the alveolar BMD around the areas of the tooth root and alveolar bone using 3D-μCT. The BMD of the alveolar bone was measured around the inter-tooth areas of the 1st and 2nd molars (Fig. 6B, left: [a]) and around the root trunk area of the 1st molar (Fig. 6B, right: [b]; indicated by the dotted square in the upper images of Fig. 6B). The alveolar BMD of the LTA injected group was significantly reduced by approximately 10% in both areas, indicating that the alveolar bone was resorbed due to periodontitis (Fig. 6B).

We next clarified the alveolar bone loss using quantitative methods that are frequently used in clinical diagnoses. Analyzing the length of the gingival sulcus to the alveolar bone (inter-tooth space) for alveolar bone loss (Fig. 6C, lengths [a]), and the bottom of the molar root trunk to the alveolar bone (root trunk space) of the 1st molar (Fig. 6C, lengths [b]; indicated by the white line in the upper images of Fig. 6C). Due to the vertical loss of the alveolar bone, it appeared that the inter-tooth space (Fig. 6C, [a]) and the root trunk space of the 1st molar (Fig. 6C, [b]) were increased. We found that the LTA significantly increased both in the inter-tooth space (Fig. 6C, [a]) and the root trunk space of the 1st molar (Fig. 6C, [b]); this reflected the progression of alveolar bone loss due to LTA-induced periodontitis (Fig. 6C).

## Discussion

In a healthy condition, the tooth root is embedded into a socket in the alveolar bone in periodontal tissue. Infection of mixed multiple Gram-negative bacteria resulted in alveolar bone resorption and tooth loss induced by severe inflammation in periodontal tissues. It is well-known that the major pathogens of periodontitis are dominantly Gram-negative bacteria, including *Porphyromonas gingivalis*, *Aggregatibacter actinomycetemcomitans, Tannerella forsythia* and *Prevotella intermedia*. LPS, as a ligand for TLR4, is an outer membrane component of Gram-negative bacteria and known to be a major definitive pathogenic factor of inflammatory bone resorption in periodontitis. On the other hand, Gram-positive bacteria have been known to contribute to the inflammation of the periodontal gums in the initial phase of periodontitis^18,19^; however, there was little evidence to show that these pathogens contribute to the induction of inflammatory bone resorption in the late phase of periodontitis. Since LTA is a major cell-wall component of Gram-positive bacteria, we decided to examine the effects of LTA on osteoclast differentiation, inflammatory bone resorption, and alveolar bone resorption in a mouse model of periodontitis.

In this study, we demonstrated that LTA promoted PGE_2_ production via the upregulation of the *Cox2* and *mPges1* mRNA expression in POBs, leading to PGE_2_-dependent osteoclast differentiation (Figs. 1 and 2). These data suggest that LTA promotes COX-2- and mPGES-1-mediated PGE_2_ production via NF-κB signaling, resulting in subsequent osteoclast differentiation. Indomethacin markedly suppressed LTA-induced osteoclast differentiation and calvarial bone resorption (Fig. 3). Furthermore, LTA maintained osteoclast cell survival, which was associated with the mRNA expression of *Nfatc1* and *Ctsk* in the absence of sRANKL (Fig. 4). We clarified that LTA could induce alveolar bone loss in the ex vivo model of periodontal bone resorption for periodontitis (Fig. 5). Finally, we confirmed that LTA induced alveolar bone loss in the model of in vivo periodontitis. In the 3D-μCT analysis, the quantitative analysis frequently used in clinical diagnoses showed that LTA significantly increased bone resorption, which became apparent with an increase in both the inter-tooth space and the root trunk space of the 1st molar in the alveolar bone (Fig. 6).

Bacterial LTA is known to be a natural ligand for TLR2/6^13,15^. In the present study, both osteoblasts and osteoclasts expressed the TLR2 and TLR6 mRNAs, and LTA induced osteoclast differentiation via PGE_2_ production and osteoclast cell survival. LTA-induced PGE_2_ production by osteoblasts upregulates the mRNA expression of *Rankl* via EP4-cAMP response element binding protein (CREB) signaling in an autocrine manner, since the promoter region of *Rankl* gene possess CRE^20,21^. In addition, a previous study reported that PGE_2_ downregulated the expression of osteoprotegerin (OPG), a decoy receptor for RANKL^22^. Thus, LTA-induced PGE_2_ production resulted in osteoclast differentiation. In bone marrow macrophages, LTA has been reported to increase the release of inflammatory osteoclastogenic cytokines, such as TNF-α, IL-1 and IL-6^23–26^. TNF-α directly acted on osteoclast precursor cells, leading to osteoclast differentiation via the upregulation of *Nfatc1* and *Ctsk*^27^. However, Kim et al. reported that IL-1 alone cannot induce osteoclast differentiation but has a synergic effect on RANKL-induced osteoclast differentiation with increasing *Ctsk* expression^28^. Inflammatory cytokines, including TNF-α, IL-1 and IL-6, act on osteoblasts to induce the expression of RANKL on the surface of osteoblasts, supporting osteoclast differentiation^29^. These cytokines also promote PGE_2_ secretion in osteoblastic cells^10,30,31^. We demonstrated that LTA increased PGE_2_ production and *Rankl* expression in osteoblasts (Fig. 2), but these effects were suppressed by indomethacin (Fig. 3). LTA also upregulated the mRNA expression of *Nfatc1* and *Ctsk* in osteoclasts (Fig. 4). These data suggest that TLR2 signaling activated by LTA derived from Gram-positive bacteria contribute to PGE_2_-mediated inflammatory bone resorption in periodontitis.

Gram-negative periodontal bacteria, such as *P. gingivalis,* mainly proliferated in the anaerobic environments of deep periodontal pockets, while Gram-positive bacteria broadly formed bacterial microbiota in the aerobic environment of the oral cavity^32^. In addition, multi-species bacterial plaques are formed and produce LTA^33^. Since the percentage of proliferating Gram-positive bacteria in the oral environment is higher than that of Gram-negative bacteria, the periodontal bone resorption in periodontitis induced by Gram-positive bacterial LTA may have potency to that induced by LPS. Both TLR2 and TLR4 signaling is reported to activate the myeloid differentiation primary response (MyD) 88-dependent NF-κB pathway^34^.

Recently, other aspects of TLR2 ligand-induced inflammation were reported. One study reported that the activity of LTA varied among bacterial species, LTA from *Staphylococcus aureus* and *Bacillus subtitis* clearly activated the NF-κB pathway via TLR2, while LTA from *Lactobacillus plantarum* showed less potency in activating the same pathway^35,36^. In the oral cavity, the presence of several Gram-positive bacteria has been reported, including *Streptococcus mutans, S. aureus*, *Streptococcus pneumoniae* and *Enterococcus faecalis*^37^. LTA from *E. faecalis* was found to induce inflammatory responses via the production of TNF-α and nitric oxide (NO)^38^. In contrast, LTA from *S. pneumoniae* has been shown to possess lower activity than *S. aureus* and to fail to form heterodimers of the isolated ectodomains of TLR2 with TLR1 or TLR6^12,39^. These reports suggest that some Gram-positive bacteria inhabiting the oral cavity may contribute to inflammation. Another biological aspect of the TLR2 ligand that has been reported is that the recognition of LTA requires co-receptors, such as CD36 and mannose binding lectin^15,40,41^. LTA from *S. aureus* also acted as the ligand for TLR2/6 heterodimer and promoted inflammatory cytokines, including TNF^41–43^. The present study showed that LTA from *S. aureus* also contributed to periodontal diseases, as *S. aureus* has been reported be part of the oral microbiota^37,44^. Further studies are needed to elucidate the roles of LTA from specified oral microbiota in periodontal bone resorption and to clarify the roles of LTA receptors, the signaling cascade, inflammatory cytokines, and PGE_2_ in periodontitis.

**Figure 7:**
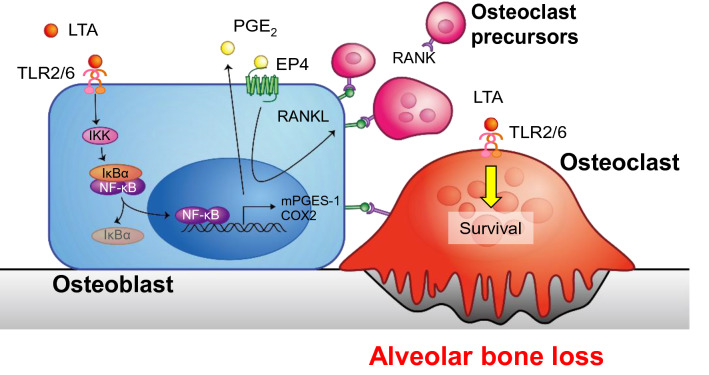
The model illustrates the effects of LTA on osteoblast and osteoclast in periodontal bone resorption. LTA induced the PGE_2_-mediated expression of RANKL in osteoblasts and directly promoted the cell survival of mature osteoclasts, leading to alveolar bone loss in periodontitis.

In conclusion, our data indicate that LTA from Gram-positive bacteria contribute to periodontal bone resorption. Figure 7 shows a schematic illustration summarizing that LTA promoted alveolar bone loss via the upregulation of PGE_2_ production and RANKL induced osteoclast differentiation and function. LTA may enhance inflammation in the early to late phases of periodontitis and periodontal bone resorption. We showed a new concept of the disease progression of periodontitis that was induced by LTA from Gram-positive bacteria.

## Materials and methods

### Animals and reagents

Newborn and 6-week-old *ddY* mice were obtained from Japan SLC Inc. (Shizuoka, Japan). All procedures were performed under animal use guidelines and approved protocol for animal research committee at the Tokyo University of Agriculture and Technology (TUAT protocol number: 29–89). LTA from *S. aureus* was obtained from Invivogen Co. Ltd. (CA, USA). Indomethacin was purchased from Fujifilm Wako Pure Chemical Corp. (Osaka, Japan). Soluble RANKL (sRANKL) was purchased from PeproTech Inc. (NJ, USA).

### Cultures of primary mouse osteoblastic cells

POBs were isolated from newborn mice calvaria after five routine sequential digestions with 0.1% collagenase (Roche Molecular Systems Inc., CA, USA) and 0.2% dispase (Roche Molecular Sytems Inc.) as previously described^1^. Collected POBs were cultured in αMEM (Thermo Fisher Scientific Inc., MA, USA) containing 10% fetal bovine serum (FBS; Nichirei Biosciences Inc., Tokyo, Japan) at 37 °C under 5% CO_2_ in air. The medium was changed twice a week.

### Osteoclast differentiation in cocultures of mouse bone marrow cells (BMCs) and POBs

BMCs were isolated from the tibiae of 6-week-old male mice. BMCs (5 × 10^6^ cells/well) and POBs (3 × 10^3^ cells/well) were co-cultured in αMEM containing 10% FBS with LTA (1, 10, 100 μg/mL) in the presence or absence of indomethacin (10 μM) in a 96-well plate with a flat bottom (AGC Techno Glass Co. LTD., Shizuoka, Japan). The medium was changed on days 3 and 5. The cells were stained for tartrate-resistant acid phosphatase (TRAP) on day 7, and TRAP-positive multinuclear cells containing 3 or more nuclei per cell were counted as osteoclasts.

### TRAP staining

Sodium acetate (0.1 M; Fujifilm Wako Pure Chemical Corp.) and 0.1 M acetate (Fujifilm Wako Pure Chemical Corp.) were mixed to prepare TRAP buffer. Naphthol AS-Mix phosphate (Sigma-Aldrich Co., LLC., MO, USA) diluted with N,N-Dimethylformamide (Fujifilm Wako Pure Chemical Corp.) and fast red violet LB salt (Sigma-Aldrich Co., LLC.) were diluted with TRAP buffer to prepare TRAP staining solution. Cells were fixed by 10% formaldehyde (Fujifilm Wako Pure Chemical Corp.) for 30 min. Fixed cells were stained by adding TRAP staining solution for 20 min.

### Bone-resorbing activity in organ cultures of calvaria

To measure the bone-resorbing activity in organ cultures of the calvaria, calvariae from newborn mice were cultured for 24 h in BGJb medium (Biggers, Gwatkin, and Judah tissue culture medium for bone; Thermo Fisher Scientific Inc.) supplemented with 1 mg/mL BSA (Sigma-Aldrich Co., LLC.) in a 24-well plate with a flat bottom (AGC Techno Glass Co., Ltd.). The calvariae were transferred to new medium in the presence or absence of LTA and cultured for another 5 days without medium exchange. The calcium concentration in the conditioned medium was measured by the *o*-cresolphthalein complexone (OCPC) method in order to determine bone-resorbing activity.

### A calcium colorimetric assay by the OCPC method

2-Aminoethanol (0.87 M; pH 11; Tokyo Chemical Industry Co. Ltd., Tokyo, Japan) was prepared as a buffer for calcium measurement. Color reagent was prepared by mixing a solution of 0.63 mM OCPC (Fujifilm Wako Pure Chemical Corp.) and 69 mM 8-Quinolinol (Tokyo Chemical Industry Co. Ltd.). Conditioned medium was mixed with 0.87 M 2-aminoethanol buffer and color reagent. The concentration of calcium was determined by the absorbance at 570 nm. Calcium solution (5, 10, 15 and 20 mg/dL) were used as standard.

### Osteoclast differentiation in cultures of Raw264.7 cells

Raw264.7 cells (4 × 10^3^ cells/well) were cultured in α MEM containing 10% FBS with or without sRANKL (100 ng/mL) for 4 days in a 96-well plate with a flat bottom. The medium was changed on day 3, and cells were continuously treated with sRANKL. After differentiation into osteoclasts in the presence of sRANKL on day 4, the medium was changed, and osteoclasts were cultured for another 1 day with or without sRANKL as well as with LTA (1, 10, 100 μg/mL) in the absence of sRANKL. Cells were stained for TRAP, and TRAP-positive multinuclear cells containing 3 or more nuclei per cell were counted as osteoclasts.

### The analysis of the mRNA expression using quantitative PCR (qPCR)

Raw264.7 cells were cultured using a similar protocol to the one described above, and total RNA was extracted. POBs (3.5 × 10^5^ cells/well) were cultured for 1 day in αMEM containing 10% FBS in a 6-well plate with a flat bottom (AGC Techno Glass Co., Ltd.). The medium was changed to αMEM containing 1% FBS, and POBs were cultured for a further day. POBs were then treated with LTA (100 μg/mL) in αMEM containing 1% FBS for 24 h. After 24 h, the total RNA was extracted from POBs. The cDNA was synthesized from 5 µg of total RNA by reverse transcriptase (Superscript II Preamplification System; Thermo Fisher Scientific Inc.). The cDNA was amplified by PCR or qPCR. The PCR primers were purchased from Eurofins Scientific SE (Luxembourg, Grand Duchy of Luxembourg), and those sequences were as follows: mouse *Actb*: (forward) 5′-ccccattgaacatggcattg-3′ and (reverse) 5′-acgaccagaggcatacagg-3′, mouse *tlr2*: (forward) 5′-cgagtggtggaagtacg-3′ and (reverse) 5′-ggtaggtcttggtgttcattatc-3′, mouse *tlr6*: (forward) 5′-ccggtggagtacctcaat-3′ and (reverse) 5′-tcagcaaacaccgagtatagc-3′, mouse *Rankl*: (forward) 5′-gagaacttgggattttgatgc-3′ and (reverse) 5′-gactccactctggagagt-3′, mouse *Cox2*: (forward) 5′-tcagccaggcagcaaatccttg-3′ and (reverse) 5′-tagtctctcctatgagtatgagtc-3′, mouse *mPges1*: (forward) 5′-atgccttccccgggcctg-3′ and (reverse) 5′-tcacagatggtgggccac-3′, mouse *Nfatc1*: (forward) 5′-agtctctttccccgacatca-3′ and (reverse) 5′-cacctcgatccgaagctc-3′, mouse *Ctsk*: (forward) 5′-cattctcagacacacaatccac-3′ and (reverse) 5′-gatactggacaccactggga-3′. The qPCR was performed with SsoAdvanced SYBR Green Supermix (Bio-Rad Laboratories Inc., CA, USA). The relative normalized expression of genes was quantified by the ΔΔCq method and β-actin was used as a normalized gene.

### Measurement of PGE_2_ production

The concentrations of PGE_2_ in the cultured medium were measured using an enzyme immunoassay (EIA) (GE Healthcare Japan Corp., Tokyo, Japan). The antibody had the following cross-reactivity determined by the bound to free ratio: PGE_2_, 100%; PGE_1_, 7.0%; 6-keto-PGF_1α_, 5.4%; PGF_2α_, 4.3%; and PGD_2_, 1.0%.

### Western blotting

POBs (8.5 × 10^5^ cells) were seeded into a 60-mm treated dish (AGC Techno Glass Co., Ltd.) and treated with LTA (100 μg/mL) for 1 h and lysed in cell lysis buffer containing protease inhibitor cocktail (Abcam plc, Cambridge, UK) and phosphatase inhibitor cocktail I (Abcam plc.). The cell lysates were centrifuged at 12,000 × *g* for 10 min, and supernatants were collected. The protein concentration of the supernatant was determined by the Bradford method. Samples (10 μg of protein) were applied to SDS-PAGE (10% polyacrylamide gel) and transferred onto polyvinylidene difluoride (PVDF) membranes. Membranes were blocked with 5% skim milk in PBS-T (PBS with 0.05% Tween-20) and incubated with primary antibodies at 4 °C. Membranes were incubated with the corresponding secondary antibody in 1% skim milk in PBS-T and developed with ECL prime western blotting detection reagent (GE Healthcare Japan Corp., Tokyo, Japan) by ChemiDoc XRS + (Bio-Rad Laboratories Inc., CA, USA) after washing three times with PBS-T. The exposure time was 10 s for β-actin and 30 s for IκBα. The membrane was adjusted to the proper size for the blotting at 25–75 kDa before incubation the primary antibody against the target protein. Blot images cropped the appropriate size for the construction of Fig. 2C. Full-length blot images and blot images in the replicated experiment were also shown in supplemental figures 1 and 2. Antibodies against IκBα (35–41 kDa) and β-actin (43 kDa) were purchased from Santa Cruz Biotechnology (TX, USA). The blots were measured intensities by Image Lab Software version 5.0 (Bio-Rad) to compare the presence of β-actin and IκBα blotted bands.

### A NF-κB dual-luciferase reporter gene assay

The pNFκB-TA-Plasmid (0.4 μg) contained four tandem copies of the NF-κB consensus sequence with the firefly luciferase reporter gene (Clontech Laboratories, Inc., CA, USA) and the pGL4.74[hLuc/TK] plasmid (40 ng) contained the renilla luciferase reporter gene (Promega Corp., WI, USA) as an internal control reporter. Both plasmids were transfected into POBs in cultures using Lipofectamine 2000 (Thermo Fisher Scientific Inc.) and cultured for 24 h with or without LTA. The luciferase activity was measured using the Dual-luciferase Reporter Assay System (Promega Corp.) with an ARVO MX multilabel/luminescence counter (Perkin Elmer Corp., MA, USA).

### Ex vivo mouse model of periodontal bone resorption and in vivo mouse model of periodontitis

Ex vivo mouse model of periodontal bone resorption and in vivo mouse models of periodontitis have been established and previously described^1^. In the ex vivo model of periodontal bone resorption, teeth extracted mouse mandibular alveolar bones were cultured for 1 day in BGJb medium containing 1 mg/mL BSA. Then, alveolar bone was transferred to new medium with LTA and cultured for 5 days. The calcium concentration in the conditioned medium was measured by the OCPC method to determine the bone-resorbing activity.

In the in vivo mouse model of periodontitis, 250 μg/mouse of LTA dissolved in PBS was injected into the left outer gingiva of the first molar in the lower jaw on days 0, 2 and 4 in 6-week-old female mice. PBS was injected into the lower gingiva at each time point as a control. Mice were anesthetized via 10 mL/kg intraperitoneal administration of three types of mixed anesthetic agents: medetomidine hydrochloride (Nippon Zenyaku Kogyo Co., Ltd., Fukushima, Japan), midazolam (Astellas Pharma Inc., Tokyo, Japan) and butorphanol (Meiji Seika Pharma Co., Ltd., Tokyo, Japan). The alveolar bone of the lower jaw was collected from mice and subjected to μCT on day 7. After μCT, the teeth were removed from the alveolar bone. The bone mineral density (BMD) of the alveolar bone with the teeth removed was measured by DXA.

### An μCT analysis and the diagnosis of the mouse model of periodontitis

For a further analysis of alveolar bone using three-dimensional (3D) reconstruction images were obtained by micro-computed tomography (μCT) (inspeXio SMX-90CT, Shimadzu, Kyoto, Japan). The bone mass was measured on a 3D image of the inter-tooth areas of the alveolar bone between the 1st and 2nd molars and the root trunk of the 1st molar. The inter-tooth spaces between the 1st and 2nd molars and the root trunk space of the 1st molar were measured on a 3D image of the alveolar bone, respectively^17^.

### Statistical analyses

All data were expressed as the mean ± standard error of mean (SEM). Student’s *t*-test was used to compare two independent groups with equal variance. Welch’s *t*-test was used to compare two independent groups with unequal variance. A one-way ANOVA, followed by Tukey’s test for post hoc analysis was used for comparisons among three or more groups. All statistical analyses were performed using the IBM SPSS Statistics software program (Ver. 25; Armonk, NY, USA).

## Supplementary Information

Supplementary Information.
