# Supplementary material for: Gram-positive bacteria cell wall-derived lipoteichoic acid induces inflammatory alveolar bone loss through prostaglandin E production in osteoblasts
Source: Sci Rep. 2021 Jun 25;11:13353. doi: 10.1038/s41598-021-92744-5 (PMC8233430; doi:10.1038/s41598-021-92744-5)
Supplement: Supplementary file 1 — Supplementary Information. [file 41598_2021_92744_MOESM1_ESM.pdf]

Supplemental figure 1

Original blots shown in Figure 2C

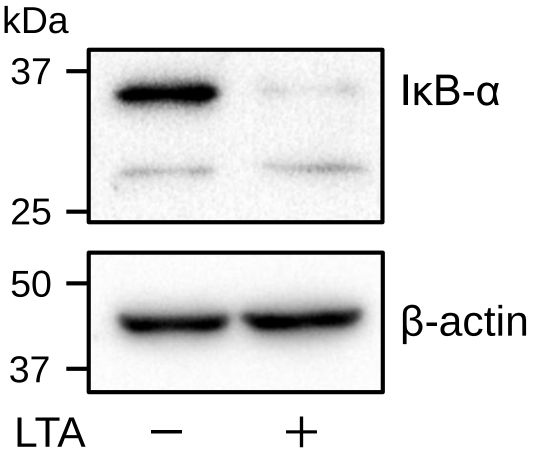

IκB-α (35-41 kDa)

β-actin (43 kDa)

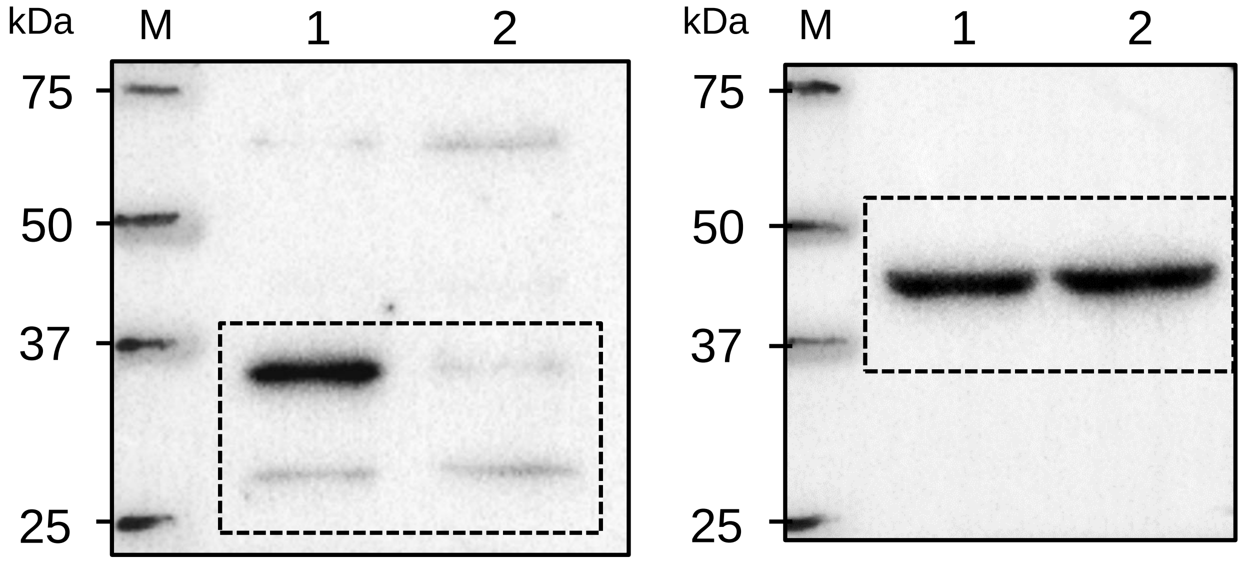

M: protein marker 1: Control group 2: LTA group

Original blots merging protein marker for the images shown in Figure 2C. Membrane was cut at 25~75 kDa prior to incubation of primary antibody against target protein.

The cropped images are highlighted in the black dotted square.

Supplemental figure 2

Blots in replicated experiment

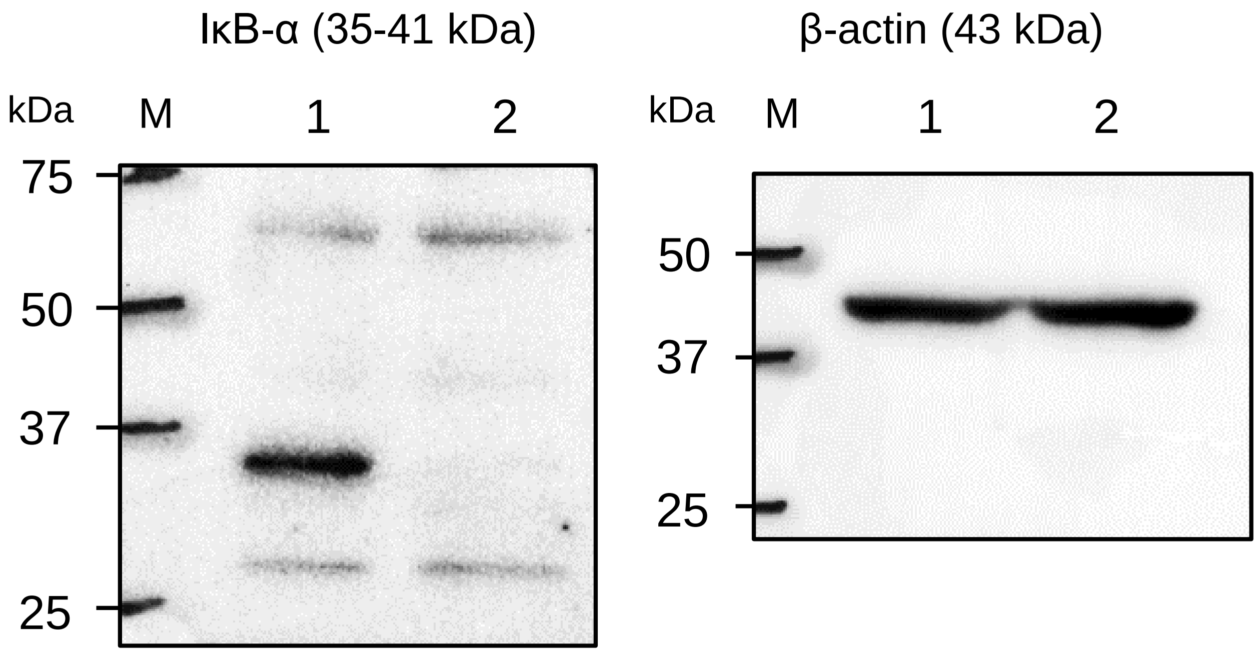

M: protein marker 1: Control group 2: LTA group

Replicated blot images merging protein marker #2 for the images shown in Figure 2C. Membrane was cut at 25~75 kDa for IκBα and 25~60 kDa for β-actin prior to incubation of primary antibody against target protein.
